# Electrospun Perovskite Nanofibers

**DOI:** 10.1186/s11671-017-1856-8

**Published:** 2017-02-13

**Authors:** Dongsheng Chen, Yanyan Zhu

**Affiliations:** 1 College of Mathematics and Physics, Shanghai University of Electric Power, Shanghai, 200090 China; 2 Department of Materials Science, Fudan University, Shanghai, 200433 China

**Keywords:** CH_3_NH_3_PbI_3_, Nanofibers, Electrospinning, Dye-sensitized solar cells

## Abstract

CH_3_NH_3_PbI_3_ perovskite nanofibers were synthesized by versatile electrospinning techniques. The synthetic CH_3_NH_3_PbI_3_ nanofibers were characterized by X-ray diffraction, scanning electron microscopy, thermogravimetric analysis, and photoluminescence. As counter electrodes, the synthesized nanofibers increased the performance of the dye-sensitized solar cells from 1.58 to 2.09%. This improvement was attributed to the enhanced smoothness and efficiency of the electron transport path. Thus, CH_3_NH_3_PbI_3_ perovskites nanofibers are potential alternative to platinum counter electrodes in dye-sensitized solar cells.

## Background

Organic-inorganic hybrid materials have useful properties, such as plastic mechanical properties and good electronic mobility. Among these materials, semiconducting CH_3_NH_3_PbI_3_ nanofibers attract considerable attention [1]. These nanofibers are currently being applied in sensitizers, hole-transporters, and combined absorber and electron transporter of nanostructured solar cells [1–3]. Spin-coating and vapor-assisted solution processes are often performed to produce perovskite thin films, and nontemplate synthesis of CH_3_NH_3_PbBr_3_ perovskite nanoparticles is already reported [4–6]. Notably, perovskite-based hybrid solar cells exceed 15% efficiency with high reproducibility [2, 7].

Currently, dye-sensitized solar cells (DSSCs) have been extensively studied because of their availability and low cost [8]. In DSSCs, the counter electrode is usually platinum (Pt), which has excellent stability with regard to its catalytic activity. However, Pt is extremely expensive and has low abundance, and thus cannot be used in large-scale commercial applications [9].

One-dimensional (1D) nanofibers have attracted considerable attention because of their large length-to-diameter ratio, high surface area, excellent aspect ratio, and effective electronic properties [10]. Through the electrospinning technique, 1D nanofibers, such as TiO_2_, ZnO, and Cu_2_ZnSnS_4_ (CZTS, 1.5 eV), can be used in solar cells [11–14]. Moreover, CZTS nanofibers can be used as replacements for counter electrodes to increase conversion efficiency [14]. Notably, the band gap and conductive type of CH_3_NH_3_PbI_3_ perovskites are similar to those of CZTSs. Thus, we explore the use of CH_3_NH_3_PbI_3_ perovskite nanofibers as counter electrodes in DSSCs.

As far as we know, we are the first to fabricate CH_3_NH_3_PbI_3_ perovskite nanofiber using an electrospinning technique. We used polyvinylpyrrolidone (PVP) as an electrospinning medium. Subsequently, to explore the application of the synthesized nanofibers, we attempted to use them as a replacement for Pt in DSSCs.

## Methods

### Preparation of CH_3_NH_3_PbI_3_ nanofiber

**Fig. 1:**
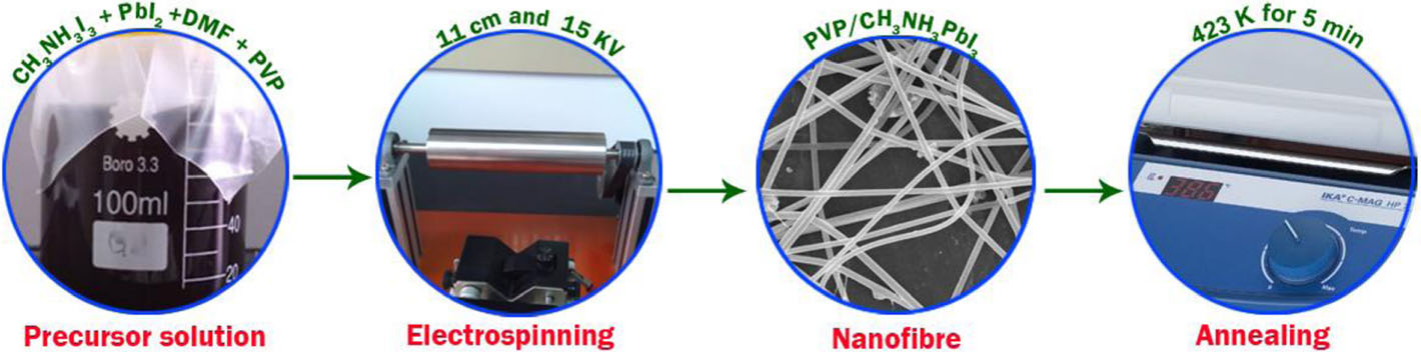
Schematic diagram of the experimental procedure for CH_3_NH_3_PbI_3_ nanofiber preparation

CH_3_NH_3_PbI_3_ precursor was prepared by adding CH_3_NH_3_I_3_ and PbI_2_ at a ratio of 1:1 to 2 mL N,N-dimethylformamide (DMF) (J&K Scientific Ltd.). Approximately 0.25 g of PVP (K90, MW = 130000) was then dissolved into the solution. The resulting solution was stirred for 30 min until a homogeneous mixture was obtained. Facile electrospinning technique method was used to fabricate the PVP-CH_3_NH_3_PbI_3_ perovskite nanofibers. The experimental procedure is schematically illustrated in Fig. 1. In the electrospinning process, the solution was injected through a stainless steel needle, which was connected to a high-voltage DC power supply. The solution was continuously fed through the nozzle using a syringe pump (LongerPump,TJ-3A/W0109-1B) at a rate of 10 μL/min. High voltage (15 kV) was applied between the needle and the grounded collector, which was situated 11 cm below the needle. As a result, a continuous stream was ejected from the nozzle and formed long fibers, which were subsequently collected. The CH_3_NH_3_PbI_3_ nanofibers obtained were calcined at 150 and 200 °C separately in a nitrogen atmosphere for 5 min.

### Characterization and Measurement

The phase structures of the prepared samples were characterized using an X-ray diffractometer (XRD, Bruker AX D8 Advance). Field-effect scanning electron microscope (Hitachi S-520) and transmission electron microscope were then used to study the microstructure of the samples (Tecnai G^2^ F20, USA). Thermal analyses were performed through thermogravimetric analysis (TGA, model Perkin Elmer Pyris Diamond) in a nitrogenous atmosphere. The band gap was measured through the photoluminescence (PL) spectrum.

## Results and Discussion

### Phase Structures

**Fig. 2:**
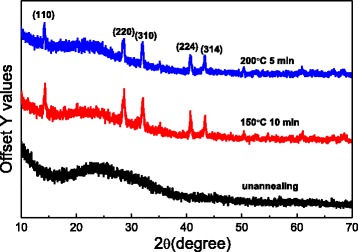
XRD patterns of the CH_3_NH_3_PbI_3_ nanofiber at different annealing temperatures

Figure 2 shows the comparison between the XRD patterns of the CH_3_NH_3_PbI_3_ perovskite nanofiber before annealing and those after annealing. Before annealing, the synthetic nanofiber is amorphous. After the 5-min annealing treatment at 150 °C, the nanofibers are able to crystallize, and the main characteristic peaks are observed at 14.04°, 28.42°, 31.76°, 40.46°, and 43.02°, which correspond to the reflections from the (110), (220), (310), (224), and (314) crystal planes of the tetragonal perovskite structure. This result is consistent with the previously published results [15]. The best annealing temperature is 150 °C. When the temperature is further increased, the main characteristic peaks are slightly reduced.

### Morphology

**Fig. 3:**
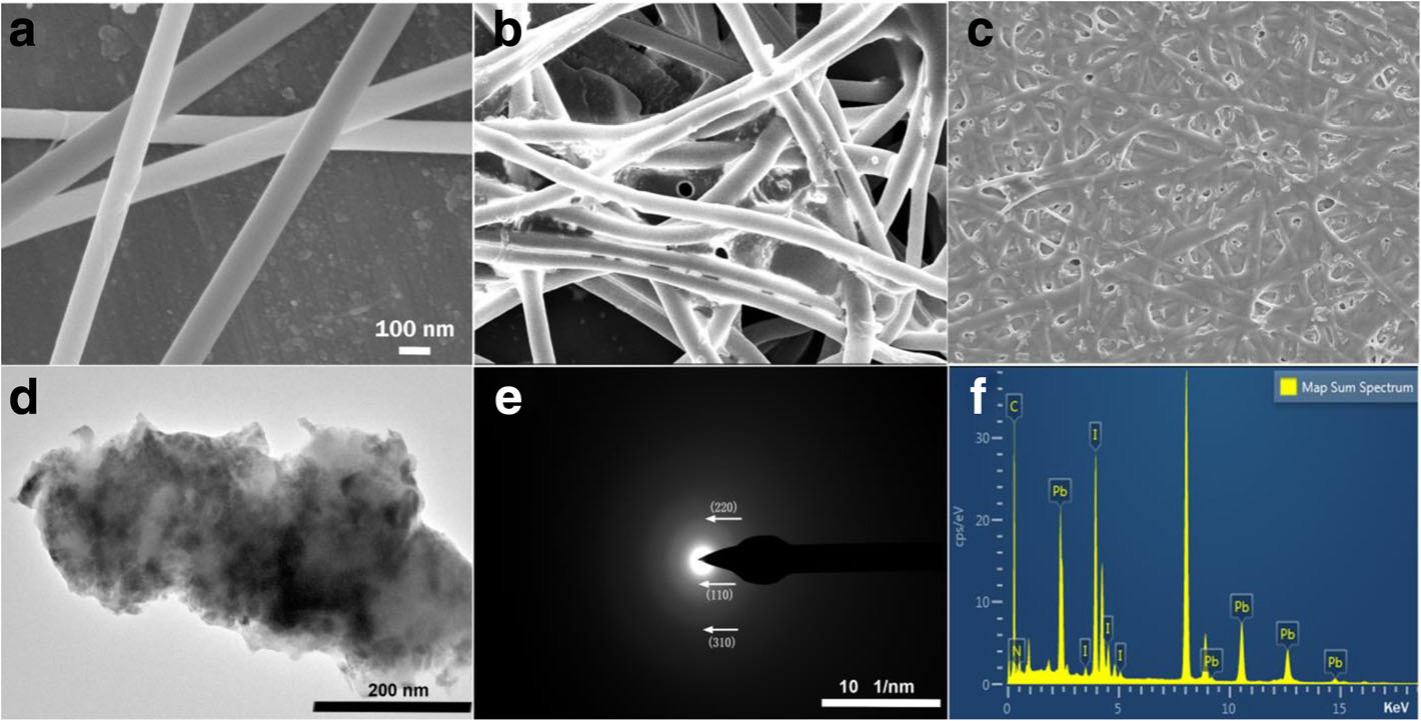
**a** SEM images of the CH_3_NH_3_PbI_3_ nanofiber, **b** and **c** SEM image after the 150 °C and 200 °C annealing treatments, **d** transmission electron microscopy image after the 150 °C annealing treatment, **e** SAED image, and **f** the EDS graph

Figure 3a–c show the scanning electron microscopy (SEM) images of the CH_3_NH_3_PbI_3_ perovskite nanofibers at different annealing temperatures. Figure 3a shows a wire-like network of CH_3_NH_3_PbI_3_ perovskite nanofibers, which are covered throughout the surface. Moreover, the surfaces of CH_3_NH_3_PbI_3_ nanofibers are quite smooth and their diameters ranges from 140 to 170 nm. Figure 3b indicates that the surfaces of the nanofibers roughened, and the diameters of the nanofibers are reduced when the nanofibers are annealed at 150 °C. When the annealing temperature is increased from 150 to 200 °C, the mesh nanofibers start to fracture and partly overlap one other (Fig. 3c). In addition, the structural features of the nanofibers exhibit a porous structure on the nanofiber wall, which contribute to the insertion of ion and facilitate its exit in the electrode material. Figure 3d indicates uniform nanocrystals. The selected area electron diffraction (SAED) image (Fig. 3e) exhibits diffraction rings corresponding to the (110), (220), and (310) directions of the CH_3_NH_3_PbI_3_ nanofibers. The appearance of multiple diffraction rings is due to the random orientation of the polycrystallites. The spotty ring pattern with missing periodicity is due to the random orientation of the particles [16]. The results of the energy-dispersive (EDS) spectroscopy on the CH_3_NH_3_PbI_3_ nanofibers after annealing treatment at 150 °C are shown in Fig. 3f. The nanofibers contain carbon, nitrogen, iodine, and lead, and have no impurity element. The compositions of the nanofiber are provided in the local compositions of C:N:H:Pb:I (0.9:0.8:4.5:1:2), which is extremely close to the stoichiometric CH_3_NH_3_PbI_3_ perovskite.

### Thermal Analysis

**Fig. 4:**
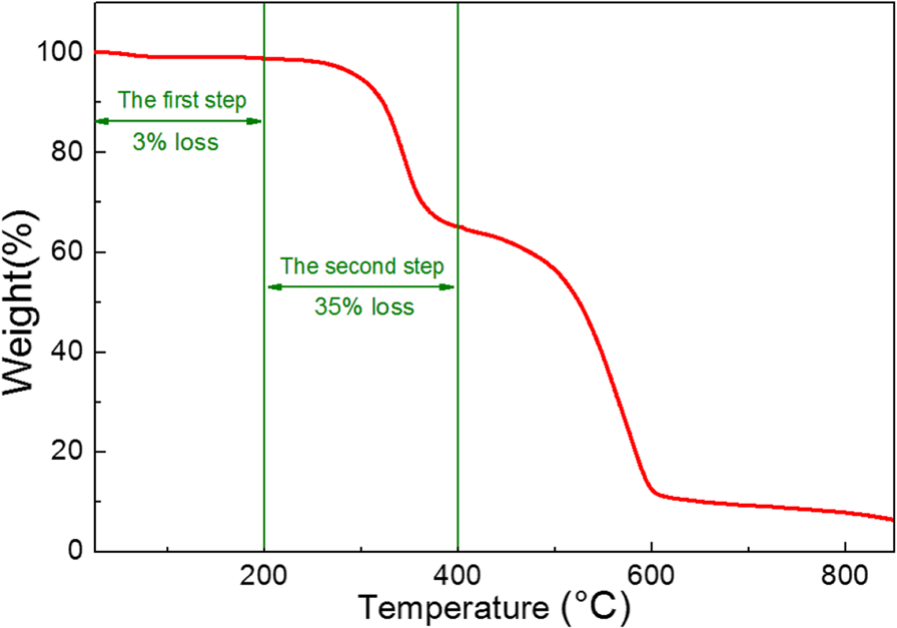
TGA curves of the CH_3_NH_3_PbI_3_ perovskite nanofibers after annealing at 150 °C

The TGA curves are shown in Fig. 4. The TGA curves have a heating rate of 20 °C/min under a N_2_ atmosphere. The curve indicates that the thermal decomposition for the as-spun nanofibers is completed in two distinct steps. In the first step, the weight loss (3%) is observed between 25 and 200 °C. The loss is due to the evaporation of water and alcohol. In the second step, (200–400 °C), the weight loss is approximately 35% and is due to PVP degradation, which involves intra- and intermolecular transfer reaction mechanisms.

### PL Spectroscopy

**Fig. 5:**
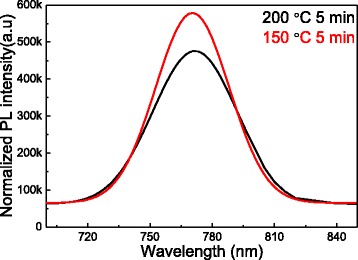
PL spectrum of CH_3_NH_3_PbI_3_ nanofibers

The band gap of the semiconductor plays an essential role in counter electrode of the DSSCs [9]. The PL spectra of CH_3_NH_3_PbI_3_ nanofiber at different annealing temperatures is shown in Fig. 5. The peak is located mainly at 770 nm, indicating that the band gap is approximately l.61 eV, which is near the absorption band edge [17, 18]. At increasing temperature, the magnitude of the PL declines because of the increased fraction of the excitonic recombination. PL quenching is expected to originate from the charge-carrier extraction across the interface [19–21]. An efficient PL quenching indicates that the charge-carrier diffusion length inside the CH_3_NH_3_PbI_3_ layer is comparable to the thickness of the layer [22].

### Application of Counter Electrodes on the DSSCs

**Fig. 6:**
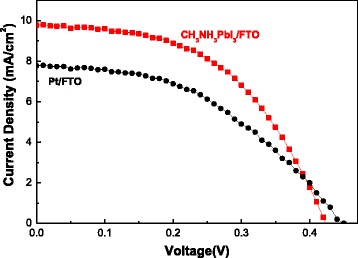
*J* − *V* characteristics in DSSCs with different counter electrodes

The CH_3_NH_3_PbI_3_ nanofiber has been applied as a counter electrode in DSSCs. The DSSCs are each equipped with a TiO_2_/FTO working electrode, redox couple (I^−^/I^3−^), and CH_3_NH_3_PbI_3_/FTO counter electrodes [14]. The current-voltage (J − V) characteristics are measured using Pt/FTO and CH_3_NH_3_PbI_3_/FTO counter electrodes. Figure 6 and Table 1 demonstrate that the open circuit voltage (*V*_OC_), short circuit current density (*J*_SC_), and filled factor (FF) of the device are 0.45 V, 7.78 mA/cm^2^, and 45%, respectively. When the counter electrodes have been changed from Pt/FTO to CH_3_NH_3_PbI_3_/FTO, the *J*_SC_ and FF values have increased slightly by 9.81 mA/cm^2^ and 51%, respectively. Moreover, the conversion efficiency of the device has increased from 1.58 to 2.09% because of the improved efficiency of the electron transport path in the CH_3_NH_3_PbI_3_/FTO electrodes. Meanwhile, the nanofibers with large surface areas contribute to the redox reaction between the counter electrode and the electrolyte, and thus, a decreased interfacial recombination in the DSSCs is observed [14].

**Table 1:** Photovoltaic performances of the DSSCs with different counter electrodes

The electrodes	Voc (V)	*J*_sc_ (mA/cm^2^)	FF (%)	Eta (%)
Pt/FTO	0.45	7.78	45	1.58
CH_3_NH_3_PbI_3_/FTO	0.42	9.80	51	2.09

## Conclusions

In summary, we successfully synthesized CH_3_NH_3_PbI_3_ nanofibers with diameters ranging from 140 to 170 nm via electrospinning technique. The XRD analysis results revealed that the synthetic nanofibers contained the pure phase of CH_3_NH_3_PbI_3_ perovskites with good crystallinity. The PL properties demonstrated that the nanofibers have a band gap energy of approximately 1.6 eV. When the nanofibers were used to the counter electrodes of the DSSCs, the conversion efficiency of the device increased from 1.58 to 2.09% because of the large surface area of the small nanofibers. Thus, our synthetic method can significantly contribute to low-cost and large-scale preparation of nanofibers for actual photovoltaic applications.

## Abbreviations

DMF: N,N-Dimethylformamide
PL: Photoluminescence
PVP: Polyvinylpyrrolidone
SAED: Selected area electron diffraction
SEM: Scanning electron microscopy
TGA: Thermogravimetric analysis
XRD: X-ray powder diffraction
